# Improved Therapeutic Delivery Targeting Clinically Relevant Orthotopic Human Pancreatic Tumors Engrafted in Immunocompromised Pigs Using Ultrasound-Induced Cavitation: A Pilot Study

**DOI:** 10.3390/pharmaceutics15061585

**Published:** 2023-05-24

**Authors:** Khan Mohammad Imran, Benjamin Tintera, Holly A. Morrison, Juselyn D. Tupik, Margaret A. Nagai-Singer, Hannah Ivester, McAlister Council-Troche, Michael Edwards, Sheryl Coutermarsh-Ott, Christopher Byron, Sherrie Clark-Deener, Kyungjun Uh, Kiho Lee, Paul Boulos, Cliff Rowe, Christian Coviello, Irving C. Allen

**Affiliations:** 1 Graduate Program in Translational Biology, Medicine and Health, Virginia Polytechnic Institute and State University, Roanoke, VA 24061, USA; 2 Department of Biomedical Sciences and Pathobiology, Virginia-Maryland College of Veterinary Medicine, Blacksburg, VA 24061, USA; 3 Department of Small Animal Clinical Sciences, Virginia-Maryland College of Veterinary Medicine, Blacksburg, VA 24061, USA; 4 Department of Large Animal Clinical Sciences, Virginia-Maryland College of Veterinary Medicine, Blacksburg, VA 24061, USA; 5 Division of Animal Science, College of Agriculture Food and Natural Resources, University of Missouri, Columbia, MO 65211, USA; 6 OxSonics Therapeutics, Oxford Science Park, Oxford OX4 4GA, UK

**Keywords:** pancreatic cancer, paclitaxel, gemcitabine, cetuximab, drug delivery, SonoTran Particles, sonoporation, passive acoustic mapping, large animal cancer model, focused ultrasound

## Abstract

Pancreatic tumors can be resistant to drug penetration due to high interstitial fluid pressure, dense stroma, and disarrayed vasculature. Ultrasound-induced cavitation is an emerging technology that may overcome many of these limitations. Low-intensity ultrasound, coupled with co-administered cavitation nuclei consisting of gas-stabilizing sub-micron scale SonoTran Particles, is effective at increasing therapeutic antibody delivery to xenograft flank tumors in mouse models. Here, we sought to evaluate the effectiveness of this approach in situ using a large animal model that mimics human pancreatic cancer patients. Immunocompromised pigs were surgically engrafted with human Panc-1 pancreatic ductal adenocarcinoma (PDAC) tumors in targeted regions of the pancreas. These tumors were found to recapitulate many features of human PDAC tumors. Animals were intravenously injected with the common cancer therapeutics Cetuximab, gemcitabine, and paclitaxel, followed by infusion with SonoTran Particles. Select tumors in each animal were targeted with focused ultrasound to induce cavitation. Cavitation increased the intra-tumor concentrations of Cetuximab, gemcitabine, and paclitaxel by 477%, 148%, and 193%, respectively, compared to tumors that were not targeted with ultrasound in the same animals. Together, these data show that ultrasound-mediated cavitation, when delivered in combination with gas-entrapping particles, improves therapeutic delivery in pancreatic tumors under clinically relevant conditions.

## 1. Introduction

Diagnosis of pancreatic cancer is associated with a poor prognosis, with a five-year survival rate as low as 6%. This poor prognosis can be attributed to the delayed presentation of patients with pancreatic cancer due to typically benign, or even a complete lack of symptoms during early stages. Location of the primary tumor can dictate the timing, signs and symptoms of pancreatic cancer, ascribing a more favorable prognosis to tumors causing symptoms in their earlier stages [1]. At this time, complete surgical resection is the only potentially curative treatment for pancreatic cancer; however, many patients do not qualify for tumor resection. Pancreatic cancers are assessed and categorized on a spectrum of high resectability to low resectability/unresectable. Characteristics of the primary tumor such as no arterial or venous involvement and no distant metastases could indicate a primary tumor with high resectability, while metastasis to lymph nodes excluding those that drain the peripancreatic tissues or involvement of the inferior vena cava are typically unresectable [2]. At the time of diagnosis, only 15–20% of patients with pancreatic cancer have tumors that are resectable due to the majority of patients having locally advanced or metastatic disease [2,3]. Chemotherapy and/or radiotherapy is currently considered to be adjuvant therapy to surgery in patients with borderline resectable tumors or as the first line treatment for patients with metastatic disease [4,5]. Although tumors characteristically have leaky blood vessels, pancreatic tumors are typically considered to be poorly vascularized and have a large amount of extracellular matrix, higher interstitial fluid pressure inside the tumor, and a lack of convection, which can all impede drug delivery to the tumor [6,7,8]. In addition to more traditional chemo- and small molecule therapeutics, monoclonal antibodies have shown promise in treating a variety of solid tumors [9,10]. However, the large molecular size of these antibodies can hinder their ability to penetrate pancreatic tumors and the use of these antibody therapies has yet to be optimized [7]. In pancreatic cancer, chemo-resistance, radio resistance and immunosuppression are all additional concerns that hinder otherwise promising therapeutic approaches [11,12,13,14]. In summary, strategies that improve the delivery and distribution of therapeutics to the tumor may significantly impact clinical outcomes [15].

Focused Ultrasound (FUS) has emerged as a noninvasive and drug-agnostic approach with the potential to improve therapeutic delivery in a variety of applications. Indeed, ultrasound-induced cavitation is an emerging approach that has shown significant promise in pre-clinical cancer studies. Of specific relevance to pancreatic cancer and the stromal niche, the use of low-intensity ultrasound combined with the co-administration of cavitation nuclei has been shown to increase drug delivery to neoplastic masses that are traditionally characterized by limited drug diffusion from the bloodstream [16,17,18,19]. For example, a mouse model using contralateral, flank-engrafted HT-29 colorectal carcinoma cells revealed significantly increased tumor concentrations of the therapeutic antibody Cetuximab [20]. Cetuximab was co-administered with cavitation nuclei consisting of either ultrasound contrast agent (SonoVue) or gas-stabilizing particles (SonoTran Particles) [20]. Ultrasound resulted in cavitation that increased tumoral Cetuximab concentrations either 2.1-fold (SonoVue) or 3.6-fold (SonoTran Particles) in US-treated tumors compared to the untreated contralateral tumor [20]. Complementing these studies, pre-clinical large animal studies have found that the SonoTran Particles combined with a clinically ready therapeutic ultrasound system (SonoTran System) are safe and effective [21] and that FUS-induced cavitation caused minimal levels of tissue damage in tissues such as the liver [22,23,24]. Thus, the objective of the current work is to expand upon these prior studies and evaluate the effectiveness of the SonoTran Particles combined with SonoTran System in a clinically relevant large animal model of pancreatic cancer that better recapitulates the human patient and tumor.

The overwhelming majority of preclinical animal models of cancer are rodent based. While mice are a valuable resource for understanding basic cancer biology and gaining mechanistic insight, due to their small size and differences in anatomy and physiology compared to human patients, direct therapeutic translation is often limited. This is especially true in the development and testing of biomedical devices, which often require multiple, miniaturized systems and optimization protocols that lack human relevance. To overcome these limitations, pigs are often used as human surrogates due to their similarity in size, genome, metabolism, anatomy, and physiology [25]. Indeed, for studies targeting pancreatic cancer, the pig pancreas is highly similar to the human organ, and the anatomic location presents many of the same ultrasound imaging and targeting complications that are typical challenges for human applications. However, to date, FUS applications in the pig have been generally limited to studies in the healthy pancreas and morbidity and mortality studies to demonstrate safety. While critical for proof of concept, the healthy pancreas does not effectively model or reproduce the tumor microenvironment, especially the stromal niche, which has significantly altered tissue mechanics compared to the healthy tissue [26,27,28]. In order to effectively take advantage of the strengths of the pig model and minimize the limitations, we recently developed a novel immunocompromised pig model that is receptive to xenografts with human tumors and tissues [22,23]. Using these *RAG2/IL2RG* knockout pigs, we have successfully engrafted multiple human cell lines, generating subcutaneous brain, breast, and liver tumors [22]. Of specific relevance to the present study, we have also effectively generated subcutaneous tumors using the human Panc-1 cell line, which is a common model of human PDAC [23]. These cells demonstrated 100% subcutaneous engraftment in our *RAG2/IL2RG* knockout pigs [23]. The tumors demonstrated characteristics that were indistinguishable from Panc-1 tumors propagated in immunocompromised mice and demonstrated hallmark features that were highly similar to characteristics typically observed in human patients [27].

In the current study, we expand the use of our novel *RAG2/IL2RG* knockout pigs and surgically engraft Panc-1 human PDAC cells in specific locations of the pancreas to generate orthotopic tumors. These clinically and physiologically relevant tumor models allow us to robustly evaluate the effectiveness of ultrasound-induced cavitation nuclei consisting of gas-stabilized particles (SonoTran Particles) in drug delivery to pancreatic tumors. Here, we characterize the orthotopic pancreatic tumors generated in the pigs and detail the effectiveness of the SonoTran System targeting. Using this model, we further show that cavitation increases the pancreatic tumor concentration of systemically delivered Cetuximab, gemcitabine, and paclitaxel. Together, these data show that noninvasive ultrasound-mediated cavitation, when delivered in combination with gas-entrapping particles, significantly improves therapeutic delivery in pancreatic tumors under clinically relevant conditions.

## 2. Materials and Methods

### 2.1. Materials

Panc-1 cells (CRL-1469) were obtained from ATCC and cultured following supplier protocols. Abraxane (paclitaxel) was obtained from Bristol Myers Squibb, New York, NY, USA. Cetuximab is a monoclonal antibody against epidermal growth factor receptor (EGFR) and was provided by Eli Lilly and Company, Indianapolis, IN, USA. Gemcitabine was obtained from Adooq biosciences LLC, Irvine, CA, USA. SonoTran Particles were manufactured by OxSonics, Oxford, UK.

The drug doses calculated for this work were extrapolated from human clinical doses. These therapeutics are administered at a dose calculated using patient surface area. To calculate a dose (mg/kg) to use in this study, for each drug, the theoretical dose for a human, minipig or micropig was calculated from literature surface area reference values. The calculated mg/kg values for a human, minipig and micro pig are shown in Table 1. Due to the increasing trend of mg/kg dose values as the recipient becomes smaller, the calculated values for micropigs were rounded upwards to provide the dosing for the immune-compromised piglet model used in this study.

### 2.2. Immunocompromised Porcine Model Generation

To generate immunocompromised pigs, we utilized the CRISPR/Cas9 system to knockout *RAG2* and *IL2RG* genes. Single-guide RNAs were designed to disrupt *RAG2* and *IL2RG* and BLASTed against the whole pig genome for their specificity. In vitro fertilization and microinjection of the CRISPR/Cas9 system were performed as previously described [22,32,33]. The immunocompromised status of each pig was validated through genotyping to confirm *RAG2/IL2RG* double knockout prior to engraftment surgery (Supplemental Figure S1). The piglets were harvested from the sow via a sterile hysterectomy, as described previously [33]. Ear notches were collected for genotyping shortly after birth. Immediately at birth, the immunocompromised piglets were aseptically transferred to their respective germ-free isolators. The pigs remained in the germ-free isolators during the course of the study to reduce the risk of infection. Each study method complied with the NIH Guide for the Care and Use of Laboratory Animals and the Virginia Tech Institutional Animal Care and Use Committee (IACUC).

### 2.3. Generation of Orthotopic Human Panc-1 Pancreatic Tumor

Human pancreatic ductal epithelial carcinoma, Panc-1 cells (ATCC, crl-1469) were cultured in RPMI media containing 10% fetal bovine serum and 1% Normocin (Invivogen) and lifted from culture plates with Trypsin (0.25%) in EDTA. Panc-1 cells were resuspended in Matrigel (Corning) on ice after trypsinization at a concentration of 6 × 10^6^ cells per 100 µL. Under general anesthesia, pigs underwent ventral midline laparotomy surgery to visualize the pancreas and engraft 6 × 10^6^ cells at two or three sites, depending on organ accessibility. The pigs resumed their regular feeding schedule at the germ-free housing facility after the surgery and post-recovery. The pigs were monitored for clinical signs of illness and tumor progression for the remaining duration of the study. Pigs were treated 26–30 days after the injection of tumor cells and the weight range of the pigs at the time of treatment was 6–7 kg. Pigs were treated as shown in Table 2, with cavitation induced in randomly selected tumors in each pig.

### 2.4. SonoTran System

The SonoTran System (OxSonics Ltd., Oxford, UK) includes an innovative hand-held curvilinear dual-frequency ultrasound probe. The probe is uniquely capable of B-mode ultrasound imaging (imaging array of 128 elements at 4 MHz central frequency), transmitting a second-frequency therapeutic ultrasound pulse (therapy array of 64 elements at 500 KHz central frequency, 8000 cycles, and 0.5 Hz Pulse Repetition Frequency (PRF)) to initiate inertial cavitation from SonoTran Particles, and, in real time, concurrently receiving acoustic emissions back from them for Passive Acoustic Mapping (PAM). These parameters have been optimized for quantifiably improved drug delivered across the vast majority of tumor models used in earlier studies [20,34,35]. PAM images correspond to the spatial images of detected acoustic energy (inertial cavitation) and is displayed as a colormap overlaid on the contemporaneous grayscale B-mode ultrasound image to localize, monitor, and achieve real-time quantification of inertial cavitation. PAM images were produced using a modified, frequency-domain Robust Capon Beamformer algorithm [36]. In order to provide real-time feedback, the calculation was implemented on a Graphics Processing Unit (GPU) using Compute Unified Device Architecture (CUDA).

### 2.5. In Vivo SonoTran Treatment

All animals were sedated using 2–4 mg/kg of Telazol-Ketamine-Xylazine (TKX) and remained under anesthesia during SonoTran treatment. Pigs were scrubbed using betadine with a special focus on the abdomen, cleaned with water, then dried, and hair covering the area of interest was trimmed using an electric trimmer. Depilatory cream was then applied to completely remove any additional hair that could hinder ultrasound imaging.

**Figure 1:**
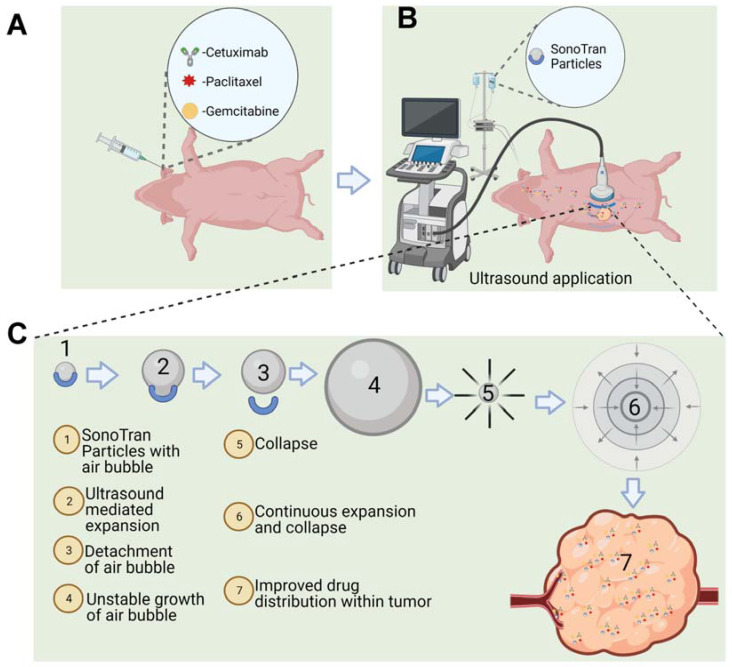
Schematic illustration of ultrasound-mediated cavitation using SonoTran Particles. (**A**) Cetuximab, paclitaxel, and gemcitabine are injected individually. (**B**) Infusion of gas-stabilized SonoTran Particles with concurrent ultrasound application targeting pancreatic tumor(s). (**C**) Schematics of air bubble expansion and collapse carried by the SonoTran Particles and FUS creating cavitation and improving drug distribution within the tumor.

SonoTran System imaging mode (B-mode) was utilized to visualize each tumor and identify appropriate acoustic windows to treat randomly selected tumors while the pigs were under anesthesia and in a supine position (Figure 1). The pigs were then injected intravenously with a bolus dose of Cetuximab (10 mg/kg), paclitaxel (5 mg/kg), and gemcitabine (40 mg/kg). The drug concentration was determined based on the clinical dose (mg/kg) for Cetuximab and paclitaxel. A lower clinical dose (40 mg/kg) of gemcitabine was used instead of a high clinical dose of 250 mg/kg. SonoTran System therapy mode was first activated to establish that the ultrasound parameters used were insufficient to induce cavitation in the absence of SonoTran Particles. The pigs were then infused with the SonoTran Particle suspension (1.48 mg/mL in 5% Glucose) at a flow rate of 3 mL per minute and mechanical pressure was applied with the probe to move bowel gas overlying the pancreas to improve visualization and cavitation monitoring. During treatment, the SonoTran System B-mode images were combined to create PAM images were used to visualize the tumor and ensure correct ultrasound-mediated cavitation targeting. The total treatment time from start of infusion of the Sonotran particles to the completion of the study was approximately 20 min. US treatment was performed immediately after (<5 min) drug injections were finished. The SonoTran Particle suspension infusion was ongoing. Each drug was administered approximately 5 min apart. Immediately post-treatment, the pigs were euthanized using euthasol intracardiac injection following our IACUC guidelines, and a necropsy was performed, gross morphology changes of organs by the treatment were recorded and organs were collected for further analysis.

### 2.6. Histopathology

Following necropsy, healthy pig pancreas, untargeted organs in the SonoTran treatment zone (i.e., gastrointestinal tract), untargeted tumors, and SonoTran targeted tumors were fixed in 10% formalin for at least 24 h prior to sectioning and staining. Tissue sections of the pig pancreas were subjected to hematoxylin and eosin (H&E) and trichrome stain to evaluate ablation, variation in collagen density and composition, damage to tumor surrounding structures (blood vessels and pancreatic ducts) in the ablation zone, and off-target damage [37]. All histopathology was evaluated by a board-certified veterinary pathologist to assess for regions of cellular damage and verify the expected degree of tissue ablation within and outside of the ablation zone.

### 2.7. Trichrome Image Scoring for Collagen Using FIJI

Trichrome-stained images were captured at 10X magnification from normal healthy pig pancreas, as well as SonoTran-treated and -untreated tumors. Images were taken from a total of 15 different representative regions of the pancreas. Images were subjected to color deconvolution into trichrome using FIJI and only the blue-stained regions (representing collagen in trichrome) were quantified. Images were converted into 8-bit images, duplicated, and thresholds were set to establish measurement parameters based on the mean and percent area, as previously described [38]. The percent area of analyzed particles in FIJI was calculated and used to generate the final data, as previously described [38].

### 2.8. Ultra-High-Performance Liquid Chromatography with Tandem Mass Spectrometry (UPLC-MS/MS)

Serum and tumor tissue was analyzed with mass spectrometry to quantify the amount of paclitaxel and gemcitabine. For mass spectrometry standards, 100 µL of serum from a healthy control pig was combined with 300 µL of the internal standard addition solution (ISAS, 1 µg/mL Gemcitabine + Paclitaxel-d5 in methanol + 1% acetic acid) in 2 mL polypropylene (PP) microcentrifuge tubes. Multiple reaction monitoring (MRM) transitions and specific mass spectrometry tuning parameters for the quantification of gemcitabine and paclitaxel are shown in Supplemental Figure S2. The protein precipitated samples were briefly shaken and then placed on a vortex table to extract for 5 min before being centrifuged (Eppendorf Microcentrifuge Model 5415R) at 16,100× *g* for 5 min. For analysis, 50 µL of the resulting supernatant solutions were then combined with 100 µL of deionized water in 2 mL amber autosampler vials with PP low-volume inserts. These were then briefly vortexed to homogenize before being placed in the refrigerated autosampler of the UPLC-MS/MS for analysis. For tissue analysis, 250 mg of tumor tissue was placed in 5 mL of acetonitrile and methanol before being mechanically homogenized. The sample was centrifuged, and the extract was removed before a secondary extraction of the homogenized tissue was performed to increase extraction efficiency. Methanol, acetic acid, and ISAS were added to the centrifuged sample post-primary extraction before being vortexed to re-homogenize prior to ultrasonic extraction for 20 min. The sample was centrifuged again at 16,100× *g* for 5 min and the supernatant was collected before being diluted, vortexed, and placed in the refrigerated autosampler of the UPLC-MS/MS for analysis. Sample extracts were subjected to chromatographic separation performed on a Waters H-Class UPLC system with an HSS T3 reverse phase column (Waters Acquity UPLC HSS T3, 100 mm length × 2.1 mm ID × 1.8 µm) and matching guard column (Waters Acquity UPLC HSS T3 VanGuard Pre-Column, 5 mm length × 2.1 mm ID × 1.8 µm) maintained at 40 °C. Four microliters of sample were injected onto the column using a refrigerated autosampler maintained at 5 °C. Mobile phase A consisted of 5 mM ammonium acetate (NH_4_CH_3_CO_2_) + 0.05% acetic acid in H_2_O, mobile phase B consisted of 5 mM ammonium formate + 0.1% formic acid in 95/5 Acetonitrile/H_2_O, and mobile phase C was 100% MeOH. The mobile phase was delivered to the UPLC column at a flow rate of 0.4 mL per min.

### 2.9. Cetuximab ELISA

ELISA was used to quantify the amount of Cetuximab, which is an antibody-based therapeutic, in each specimen. The ELISA was not specific to Cetuximab but was suitable for any human antibody in pig blood and tissue samples. An untreated tumor from a pig that did not receive Cetuximab was used as a negative control. Tumor samples were weighed and homogenized in 1× cell culture lysis reagent (Promega, Hampshire, UK) and protease inhibitor cocktail (Abcam, Cambridge, UK) at a concentration of 1.25 mg/mL. Known quantities of Cetuximab were added to this tumor homogenate and serially diluted to generate a standard curve to quantify the amount of Cetuximab in the experimental specimens. For each ELISA, 50 µL of the primary antibody (Goat anti-human IgG-Fc specific, Sigma-Aldrich, St. Louis, MO, USA) was used to coat the wells of a 96-well high binding ELISA plate at a concentration of 1 µg/mL before being incubated overnight at 4 °C. Wells were washed at room temperature with wash buffer (PBST, 0.1% Tween 20 in 1× PBS), ensuring bubbles and liquid were removed from the wells at each wash. The wells were then blocked with 200 µL of 5% BSA in 1× PBST for at least one hour. The wells were washed again with wash buffer, and 50 µL of the standards or samples was added to the plate in either triplicate or duplicate, before being left to incubate for 1.5 h. The wells were washed again with wash buffer before being incubated with goat anti-human horseradish peroxidase-conjugated secondary antibody (Promega, Hampshire, UK) diluted to 1:2500 in 5% BSA in 1× PBS for 1.5 h at room temperature. After the wells were washed three times with wash buffer, TMB substrate (ThermoFisher Scientific, Oxford, UK) was added to the wells and incubated in the dark until a deep blue color had developed in the highest concentration standards. To stop the reaction, 50 µL of 1M H_2_SO_4_ was added per well and absorbance at 450 nm was determined using a plate reader (SpectraMax M5, Molecular Devices, San Jose, CA, USA). Absorbance values from control samples were used to generate a Four Parameter Logistic (4PL) Curve in AAT Bioquest website and sample absorbance values were extrapolated from this curve.

### 2.10. Statistical Analyses

Unless otherwise noted, data were analyzed using GraphPad Prism, version 9.0. Statistical significance was indicated as *p* < 0.05. All data are denoted as the mean ± SEM (ELISA and MS data show individual animal data points). A student’s two-tailed paired t-test was performed when evaluating two experimental groups or an ANOVA was utilized with Tukey’s post-test for complex datasets.

## 3. Results

### 3.1. Orthotopic Pancreatic Tumors Were Successfully Generated in the RAG2/IL2RG Deficient Immunocompromised Pigs

**Figure 2:**
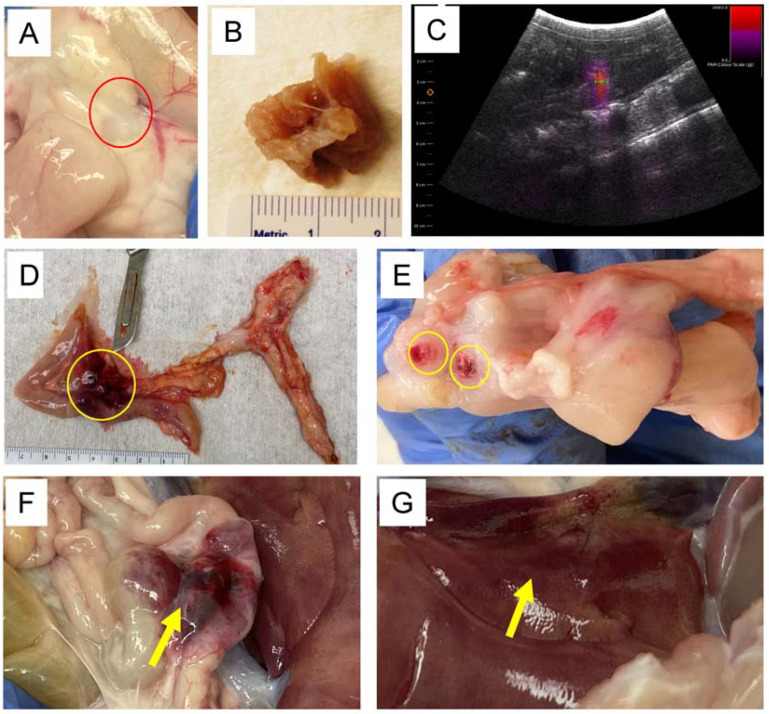
Panc-1 human pancreatic cancer cells were successfully engrafted in the pig pancreas and were effectively targeted using focused ultrasound. (**A**) Representative gross image of a human Panc-1 tumor in situ. (**B**) Excised human Panc-1 tumor. (**C**) US image of ongoing SonoTran therapy. Red color indicates active therapy targeting pancreatic tumor. (**D**) Gross image of human Panc-1 tumor grown in immunocompromised pig pancreas and hemorrhage from ultrasound-induced cavitation (indicated by yellow circle). (**E**) Gross image of Panc-1 tumor and hemorrhage following ultrasound-induced cavitation (indicated by yellow circles). (**F**) Off-target hemorrhage in the intestine (Yellow arrow). (**G**) Off-target hemorrhage in the liver (Yellow arrow).

Generation of the *RAG2/IL2RG* knockout pigs was conducted as previously described [23,24,33]. Briefly, gRNAs targeting pig *RAG2* and *IL2RG* genes and Cas9 mRNA were injected into presumable zygotes following in vitro fertilization and cultured for five days. The injected embryos were transferred to surrogate gilts. Piglets were delivered through hysterectomy into a germ-free housing system, 114 days after in vitro fertilization. A total of eight piglets were utilized for the studies described. Genomic DNA from piglet ear notches revealed that both the *RAG2* and *IL2RG* genes were successfully disrupted in all eight piglets (Supplemental Figure S1). Abdominal surgery was performed under anesthesia to visualize and inject the Panc-1 human pancreatic cancer cells into the pancreas for xenograft implant. A total of 100 µL containing 6 × 10^6^ Panc-1 cells was injected into specific regions of each pancreas, with each pig engrafted with either two or three tumors. Pigs were relocated to their germ-free isolators once they had fully recovered from anesthesia and they were clinically monitored throughout the remaining duration of the study. Every pig developed at least one tumor, with an orthotopic xenograft success rate of 94.44% (Table 2). Gross in situ tumors and excised formalin-fixed tumors are shown in Figure 2A,B respectively. Tumors were well tolerated without any illness and other abnormalities for the duration of this study and pigs were maintained for up to 45 days post-tumor engraftment, which corresponded to tumors reaching the average targeted treatment size of approximately 1 cm in diameter. The tumors were nodular and highly similar to tumors generated in immunocompromised mice, demonstrating multiple features that were consistent with tumors evaluated from human PDAC patients. The tumors were significantly stiffer than the surrounding pancreatic tissue and did not show regions of central necrosis, which is often found in mouse pancreatic tumor models.

### 3.2. FUS-Mediated Cavitation Using SonoTran Particles Effectively Targeted Orthotopic Panc-1 Tumors in the Pancreas, with Predominantly Superficial Off-Target Effects

**Figure 3:**
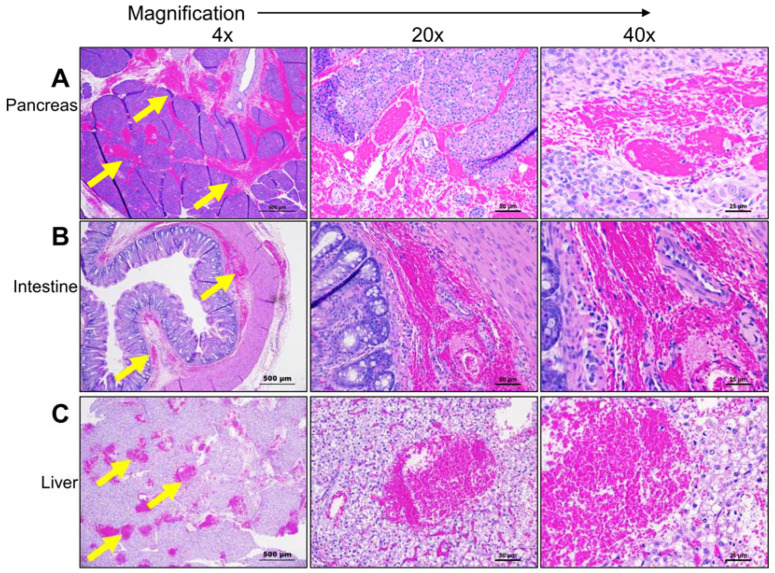
Histopathology assessments reveal ultrasound-mediated cavitation hemorrhaging in healthy organs within the treatment zone. Representative images of H&E-stained tissue sections were generated during histopathological evaluation of treatment effectiveness. (**A**) Treated immunocompromised pig pancreas showing minor to moderate hemorrhaging (yellow arrow) within the US treatment window of the healthy pancreas. (**B**,**C**) Additional off-target hemorrhaging was observed in the (**B**) intestine and (**C**) liver of treated animals. (**A**–**C**) Left to right- 4×, 20× and 40×, respectively. Scale bars are 500 µm for 4×, 50 µm for 20×, and 25 µm for 40× magnification.

**Figure 4:**
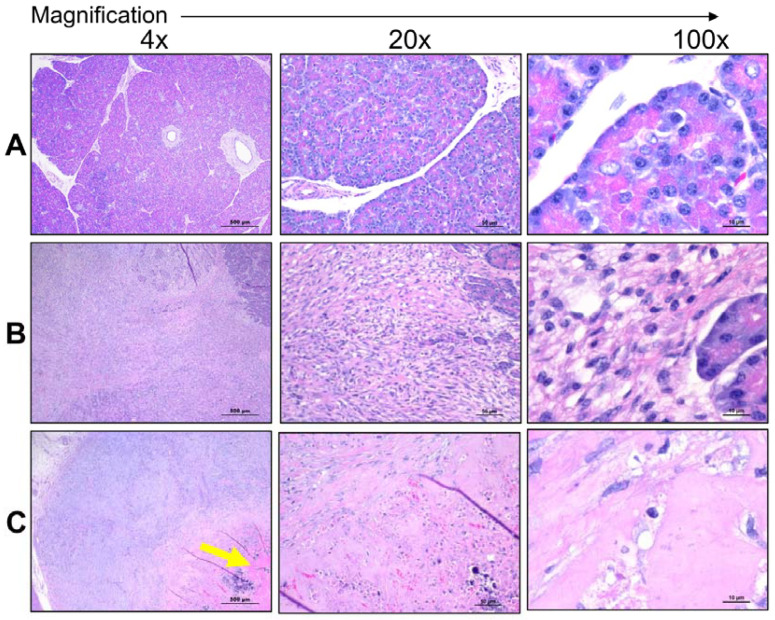
Effective targeting of human Panc-1 orthotopic porcine tumors in situ using ultrasound-mediated cavitation. Representative images of H&E-stained tissue sections were generated during the histopathological evaluation of treatment effectiveness in pigs with engrafted tumors. (**A**) Immunocompromised pig pancreas without ultrasound treatment. (**B**) Human Panc-1 tumors were effectively generated in the *RAG2/IL2RG*-deficient pigs and demonstrate histopathological features consistent with orthotopic cell line-based tumor models and human patient tumors. (**C**) Ultrasound-mediated cavitation resulted in clear margins defining the treatment zone with altered tumor structural features and areas of increased hemorrhage (yellow arrow). (**A**–**C**) Left to right- 4×, 20× and 100×, respectively. Scale bars are 500 µm for 4×, 50 µm for 20×, and 10 µm for 100× magnification.

For the current study, US was applied noninvasively through an abdominal window determined by using SonoTran System combined to SonoTran Particles. The pancreas was imaged, and tumors were detected and targeted within the ultrasound acoustic window in each pig (Figure 1 and Figure 2). Prior to SonoTran treatment, we intravenously infused the pigs with Cetuximab (10 mg/kg), paclitaxel (5 mg/kg), and gemcitabine (40 mg/kg) (Figure 1A). Drug injections were then followed by infusion with SonoTran Particles (3 mL per minute) using an infusion pump to maintain a stable infusion rate during the US application (Figure 1B,C). SonoTran treatment was performed for around 20 min, and the FUS-mediated cavitation treatment phase was visualized using PAM images (the red area corresponding to the prediction of induced cavitation; Figure 2C). For the three pigs treated, inertial cavitation was efficiently induced in the pancreas. Cavitation energy measured by PAM displayed as the maximum energy value in Joules per FUS-pulse was used as a reference to cavitation activity. These “PAM max” values are plotted against time and peak rarefactional focal pressure (PRFP) in MPa (Supplemental Figure S3). Immediately upon the conclusion of SonoTran Treatment, pigs were euthanized, and a necropsy was performed. During the necropsy, both targeted and nontargeted tissues and organs were collected for subsequent analysis. Targeted tumors were readily identified by gross pathological lesions and regions of hemorrhage, which were grossly identified on all targeted and FUS treated tumors in the pancreas (Figure 2D,E). Grossly, off-target effects were also observed, almost exclusively in regions of the gastrointestinal tract that overlaid the pancreas within the acoustic window or injury to the liver in regions that were adjacent to the targeted region of the pancreas (Figure 2F,G). While these lesions were clearly identified grossly, histopathological assessments revealed that most of the off-target damage was superficial bruising or minor hemorrhaging (Figure 3). Hemorrhage was observed histopathologically in regions of the healthy pancreas near or adjacent to the tumors (Figure 3A). We also observed histopathologic evidence of hemorrhage in regions of the small intestine, especially in areas that overlay the pancreas and were within the acoustic window, and areas of the liver that were adjacent to the pancreas near the treatment zone (Figure 3B,C). Hemorrhaging in the nontargeted regions of the pancreas, intestine, and liver generally presented as small, punctate spherical patches spread over a wide area of the tissue (Figure 3A–C). No hemorrhaging or tissue damage was observed in the pancreas or tumors from animals that received the SonoTran Particles and therapeutics, but were not targeted with the US (control tumors, Figure 4A,B).

### 3.3. Ultrasound-Induced Cavitation with SonoTran Particles Increases Drug Concentrations in Targeted Pancreatic Tumors

**Figure 5:**
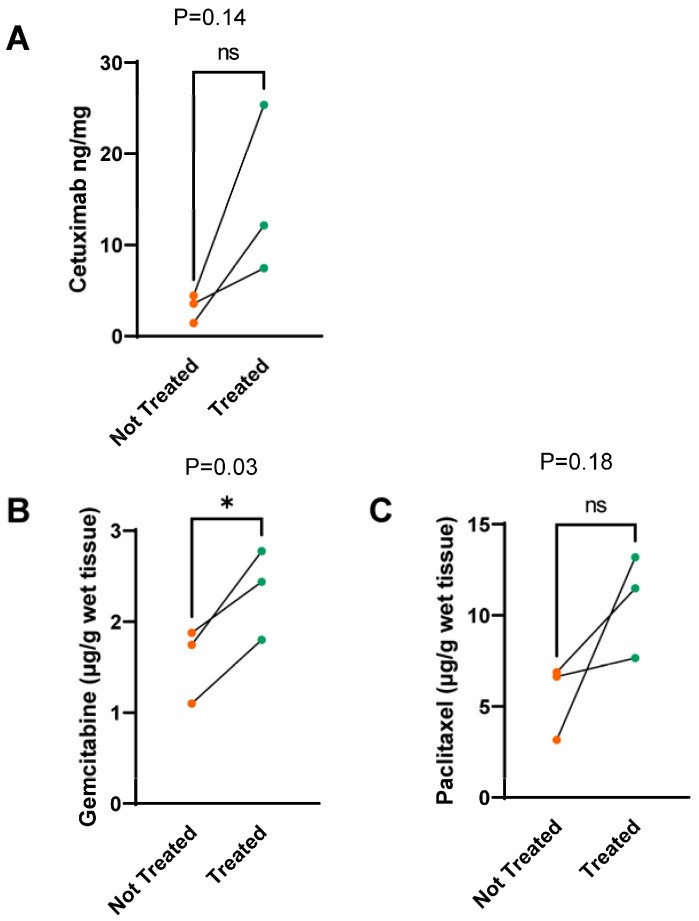
Ultrasound-mediated cavitation using cavitation nuclei increases the tumor delivery of cetuximab, gemcitabine, and paclitaxel. ELISA and mass spectrometry (UPLC-MS/MS) was used to quantify Cetuximab, gemcitabine, and paclitaxel, respectively. (**A**) ELISA quantification of Cetuximab comparing ultrasound targeted (treated) and untargeted (untreated) tumor tissue shows significant accumulation in the treated tumor. (**B**,**C**) MS analysis of gemcitabine and paclitaxel comparing treated and untreated tumor tissue reveals increased delivery of both drugs in the ultrasound-treated tumor compared to the untreated tumor. ns denotes not significant; * denotes *p* < 0.05.

Collagenous stroma is a significant factor in chemoresistance, in part, by inhibiting drug penetration within the tumor [14,39,40]. Thus, we predict that the US and SonoTran therapy would significantly improve drug delivery. To evaluate this hypothesis, we evaluated the tumor deposition of three common cancer therapeutics: Cetuximab, gemcitabine, and paclitaxel (Figure 5). Each therapeutic was individually infused, intravenously, into pigs. Upon completion of the therapeutics injection, animals were infused with the SonoTran Particles, and a simultaneously selected tumor in each animal was targeted with FUS. Upon completion of SonoTran infusion and FUS treatment, the animals were euthanized, and drug concentrations were determined in the FUS-treated and untreated tumors in each animal using either ELISA for the antibody Cetuximab (Figure 5A) or mass spectrometric analysis (UPLC-MS/MS) to quantify gemcitabine and paclitaxel (Figure 5B,C). We observed a 4.77-fold trending increase in the concentration of Cetuximab in the FUS-targeted tumors compared with the tumors that were not targeted with FUS (Figure 5A, *p* = 0.14). Likewise, we observed a significant increase in gemcitabine in the FUS-targeted tumors compared with the tumors that were not targeted with FUS, with levels increasing from 1.58 to 2.34 µg/g of wet tissue (Figure 5B, *p* < 0.05). Similar to the increased concentrations of Cetuximab and gemcitabine, we also observed a consistent increase in paclitaxel in the FUS-targeted tumors, with levels increasing from 5.57 to 10.78 µg/g of wet tissue (Figure 5C, *p* = 0.18). However, there was more variability in the paclitaxel tumors treated with FUS (Figure 5C). Thus, these data were trending but did not achieve statistical significance. Taken together, our data suggest that ultrasound-induced cavitation with SonoTran Particles increases drug concentrations in targeted pancreatic tumors.

### 3.4. FUS-Mediated Cavitation Using SonoTran Particles Increased Tissue Disruption and Reduced Stromal Collagen in the Tumor Microenvironment

**Figure 6:**
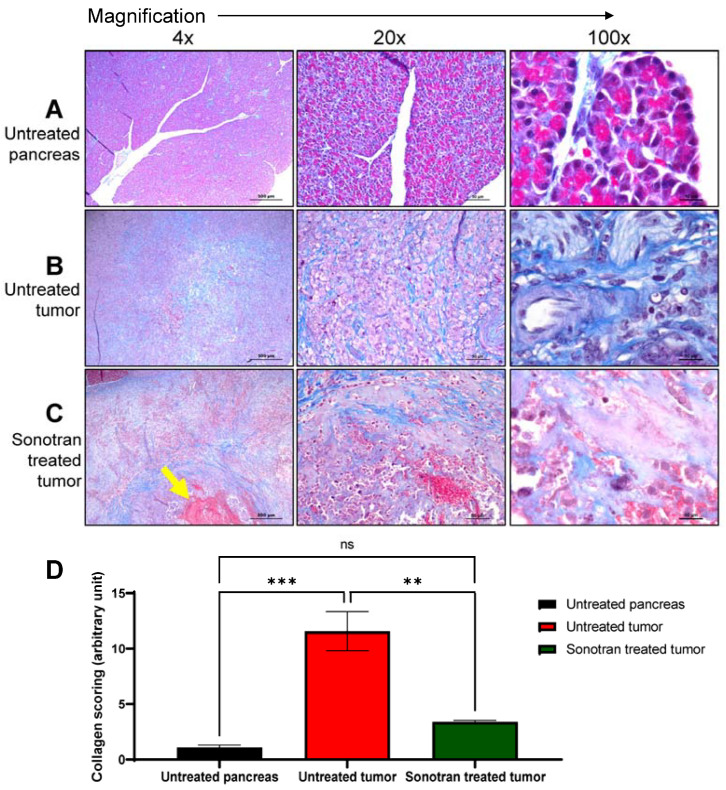
Trichrome stain reveals differences in collagen deposition and stroma among control, tumor and SonoTran-ultrasound-treated tumors. Representative tumor sections were stained with Trichrome for analysis of collagen deposition and tumor stroma assessments. (**A**) Immunocompromised pig pancreas without ultrasound treatment. (**B**) Orthotopic Panc-1 tumor in pig pancreas, without ultrasound treatment. (**C**) Panc-1 tumor treated with ultrasound-mediated cavitation using SonoTran Particles. (**D**) Quantification of trichrome images using FIJI image analysis reveals a reduction in collagen (blue staining in the images) after therapy. (**A**–**C**) Left to right- 4×, 20× and 100×, respectively. Scale bars are 500 µm for 4×, 50 µm for 20×, and 10 µm for 100× magnification. Areas of increased hemorrhage are noted with the yellow arrow. ns denotes not significant, ** denotes *p* < 0.01, *** denotes *p* < 0.001.

The dense tumor stroma is a significant inhibitor of drug delivery in many different tumor types, including pancreatic tumors. As shown in Figure 4A, the healthy pig pancreas is composed of lobules separated and defined by connective tissue septae. Lobules of the pancreas contain clusters of exocrine acini cells. In the endocrine section of the pancreas, fully vascularized areas of several cell types called Islets of Langerhans are embedded within the pancreatic exocrine tissue. Cell cytoplasm and nuclei are clearly defined histopathology assessments of H&E-stained tissue (Figure 4A). Nodular Panc-1 tumors, with clear margins, were successfully engrafted in specific regions in the pig pancreas near anatomic landmarks to facilitate ultrasound targeting (Figure 4B). Histopathological evaluation of the tissue revealed that the tumors have no distinct lobules and cells were disarrayed or had a stream-like feature (Figure 4B 100×, right panel). Hemorrhaging around and within SonoTran targeted tumors was readily observed (Figure Figure 3A and Figure 4C). Due to the mechanical force created by the continuous expansion and collapse of the bubbles stabilized in the SonoTran Particles (Figure 1C), and the application of higher cavitation energy to support increased drug delivery, we anticipated that the cavitation may reduce stromal density within treated tumors. To evaluate the effect of the therapy on the stroma density, we evaluated collagen content using trichrome staining and semi-quantification of density using FIJI image analysis (Figure 6). Consistent with pancreatic tumors in human patients, the Panc-1 tumors demonstrated a significant, 11-fold increase in collagen content compared to the normal, healthy pig pancreas (Figure 6A vs. Figure 6B, and quantification in Figure 6D). However, following FUS with SonoTran Particles, we observed a significant, 3.5-fold decrease in collagen compared to the untreated tumors (Figure 6B vs. Figure 6C and quantification in Figure 6D). Earlier studies have determined the micro-heterogeneity of collagen in human pancreatic tumors and showed that the amount of collagen is significantly associated with the biomechanical stiffness of tumors and inversely associated with vascular perfusion [37].

## 4. Discussion

Despite therapeutic progress over the last decade, pancreatic cancer has consistently ranks as one of the most lethal malignancies. A major driver of the dismal survival rate among patients is associated with the lack of effective chemotherapy treatment strategies and low response rates for highly promising immunotherapeutics. The lack of drug penetration into the solid tumor and poor drug distribution within the tumor microenvironment are typically cited as reasons for the failure of these therapeutics [7,41]. In pancreatic cancer, poor drug distribution is commonly driven by dense stroma formation and its physical/mechanical barriers. For example, the tumor interstitium can include a dense collagenous matrix that can constitute a total volume of up to 40% of the tumor [7]. These tumors can also have higher interstitial pressure inside the tumor compared to the periphery, with a lack of convection, and uneven or disarrayed distribution of vasculature-mediated blood supply can contribute to poor, nonuniform and uneven drug distribution [7,41,42,43]. Complementing these physical and environmental barriers, the development of chemoresistance is often relatively high for pancreatic cancer [26,42,43,44,45,46]. Beyond the physical complications associated with the tumor, many of the therapeutics used to treat pancreatic cancer also have significant limitations. For example, most drugs have high levels of detrimental side effects, off-target adverse events, and lower quality of life, due in part to the necessarily toxic nature of the drugs. This is partially driven by the systemic application of the therapeutics necessary to achieve the locally high doses necessary to impact tumor progression. Many of the current drugs used for pancreatic cancer also have relatively poor in vivo stability, which also necessitates high dosage ranges to compensate for drug breakdown. These issues are compounded by reduced local delivery to the tumor-associated with attenuated extravasation from the circulation and minimal penetration into the tumor, again driven in part by dense stroma formation [42,43,47]. Together, these represent significant challenges for pancreatic cancer drug delivery and refinements are critical to improving patient survival.

To address these issues, our results demonstrate that combining noninvasive focused ultrasound, coupled with co-administered cavitation nuclei consisting of gas-stabilizing SonoTran Particles, is effective at increasing local drug delivery for all three of the therapeutics tested. The greatest increase was observed for Cetuximab, for which our approach effectively doubled the local tumor concentration. Similarly, the combined use of focused ultrasound coupled with cavitation nuclei significantly increased gemcitabine, and we consistently observed a trend towards increased paclitaxel. As this was a pilot study with a limited number of animals, trending was observed and further studies are required to increase statistical power. The Cetuximab data are highly promising because it is a monoclonal antibody-based therapeutic that targets the extracellular domain of the epidermal growth factor receptor (EGFR). Prior Cetuximab studies have shown convincing results in preclinical mouse studies of pancreatic cancer [48,49]. However, the findings from human studies have been more mixed. For example, a Phase II study combining Cetuximab with gemcitabine in locally advanced or metastatic pancreatic cancer patients reported promising results [50]; however, a larger prospective phase II/III trial did not reveal any benefit or minimal benefit of Cetuximab [51,52]. Gemcitabine is one of the primary chemotherapy drugs used to treat pancreatic cancer and, as mentioned above in the Cetuximab trial, is commonly combined with other therapeutics. Of particular relevance to the current study, it is commonly administered with paclitaxel formulations, such as the Nab-paclitaxel (Abraxane^®^) combination used here. In early clinical trials, gemcitabine was proven effective as a single-agent therapeutic, improving symptoms and showing clinical benefits in approximately 20–30% of patients, with survival rates increasing by as much as 18% [53]. However, while these results are promising, the rise of chemotherapy-resistant tumors is still a significant challenge with gemcitabine, indicating the need for combination approaches [39]. Recently, clinical trials using combined gemcitabine and Nab-paclitaxel have produced impressive clinical effectiveness and safety profiles in both locally advanced and metastatic pancreatic cancer in patient populations [54]. Despite this progress, the overall survival rate for pancreatic cancer patients, especially those presenting with advanced stage, remains dismal, with poor five-year survival rates as low as 2–9% [55]. For each of these therapeutics, the suboptimal clinical effects and overall attenuated efficacy have been attributed to a combination of factors that includes high toxicity due, in part, to the elevated dosage necessary to effectively deliver the drugs to the pancreatic tumor and the molecular mechanisms of the drugs that can limit cellular uptake and tumor distribution. Based on our findings, combining ultrasound with co-administered cavitation nuclei has the potential to overcome these limitations by increasing the local concentration (i.e., drug infiltration into the tumor) of these therapeutics, thus enabling improved dosing and limiting the toxicity profiles of the therapeutics. Future studies can explore whether the cavitation-enhanced delivery of Cetuximab, gemcitabine, and paclitaxel results in improved therapeutic efficacy.

Our findings associated with improved drug delivery using ultrasound-induced cavitation are consistent with prior large-animal studies and rodent findings. Specifically related to the use of the SonoTran platform, prior studies have shown effective cavitation induction, detection, and display of cavitation events in real time using a passive acoustic mapping approach in various targeted tissues (e.g., liver) for up to one hour in large animal models [21]. These findings demonstrated the feasibility and safety of controlled cavitation in a large animal using a clinic-ready platform [21]. Expanding upon these data, a second study in rodents has also been reported, which utilized ultrasound-mediated cavitation nucleated by gas-entrapping nanoparticles, whereby the delivery of Cetuximab was found to be significantly improved in xenograft tumors [20]. Human HT-29 colorectal adenocarcinoma cells, which are strongly positive for EGFR, were engrafted bilaterally in the mouse flank and used as models to evaluate drug delivery [20]. Similar to the studies described here for the pig pancreatic cancer studies, Cetuximab was co-administered intravenously with cavitation nuclei (either SonoVue ultrasound contrast agent or SonoTran Particles) and one of the two tumors was exposed to focused ultrasound [20]. In the mouse study, cavitation increased Cetuximab concentrations either 2.1-fold with SonoVue or 3.6-fold with SonoTran Particles [20]. Together, these prior studies are highly similar to the findings we report in the current work. However, the prior studies were somewhat limited by the models utilized. For example, the prior large animal studies focused on monitoring cavitation in healthy tissues, which have vastly different physiology and mechanical properties compared to tumor tissue [22,23,24]. Likewise, the mouse studies are highly informative, albeit not highly relevant to in situ/orthotopic tumors or for direct clinical translation. Thus, the data generated here using our novel pig models with in situ relevant pancreatic tumors, surgically localized in areas of the pancreas that are difficult to both target and treat using traditional approaches, provide an ideal model for the evaluation of this drug delivery approach. In this model of human pancreatic cancer, targeted cavitation was readily observed and monitored in real time. Likewise, we observed significant increases in drug delivery in ultrasound-targeted tumors. It should be mentioned that off-target effects were clearly noted in the studies described here, especially hemorrhaging in the gastrointestinal tract that overlaid the pancreas. While this was pathologically determined to be superficial and predicted to have minimal clinical impact, the description of these events is critical for establishing the safety of the treatment strategy. It should also be noted that the tumors were treated with larger cavitation energies to maximize potential stromal penetration compared to prior treatments in large animals and mice in the described studies [20,21], which likely contributed to many of these off-target effects. Together, these studies are highly complementary to prior work in the field and further demonstrate the feasibility of using this approach to increase drug deposition in pancreatic tumors under highly relevant clinical conditions.

The effective deployment of our novel pig models described here is a significant innovation in drug delivery studies. Indeed, the lack of effective pre-clinical animal models of pancreatic cancer has contributed to the limited progress in drug development. In the biomedical device field, it is highly common to utilize pigs for the development and testing of human-relevant systems to evaluate safety and proof-of-concept studies in healthy tissues. Likewise, these studies are often complemented using mouse or rat models of either subcutaneous or orthotopic tumors and miniaturized devices with modified protocols to account for the small size of the animal models. This is particularly challenging in the mouse pancreas, which does not share many anatomical similarities with the human organ; additionally, its small size typically excludes practical orthotopic studies. Thus, the pig models deployed here minimize these limitations and provide us with a human-relevant model for therapeutic assessments. The ability to surgically engraft human tumors in the pancreas provides us with a highly robust, controllable, and reproducible model of pancreatic cancer. As noted in our results, the in situ pancreatic tumors that develop in these pigs are identical to tumors generated in orthotopic mice from Pan02 cell lines and demonstrate a variety of features that are consistent with tumors isolated from human patients [22,23,24]. While we have found this model to be ideal for biomedical device development and drug-targeting studies using human cell lines and patient-derived xenograft tumors, the lack of a functional immune system can limit some studies that require the immune niche. Beyond the pancreas, we have utilized these animals to engraft human liver, brain, breast, and sarcoma cell lines [22,23,24,56], which will be utilized in future studies to expand upon the findings related to ultrasound-induced cavitation and drug delivery.

The development of a dense collagenous stroma is one of the features of the porcine Panc-1 model that enables effective, realistic studies of drug delivery to pancreatic tumors. As described in the present work, the Panc-1 tumors demonstrate increased intra-tumoral collagen deposition, along with reduced and disarrayed vasculature (Figure 4). Following focused ultrasound treatment and cavitation, we observed significant disruption to this collagen matrix and increased hemorrhage in the targeted regions of each tumor. This is consistent with other prior studies that have reported damage to blood vessels through nanoparticle cavitation, which has been speculated to normalize the interstitial pressure inside of the tumor, leading to improved drug delivery and ultimately reducing the tumor burden [57]. Drugs can be distributed within a solid tumor through diffusion and convection. Small-molecule chemotherapeutics, such as 5-FU (130.078 Da), gemcitabine (263.198 Da), and paclitaxel (853.92 Da), enter the tumor predominantly through diffusion from the blood vessels, whereas, large molecules such as monoclonal antibodies, i.e., Cetuximab (134 kDa) predominantly enter the tumor through convection [7]. Intriguingly, mathematical models of drug infusion within solid tumors have shown that a molecule of 150 kDa would take up to a month to evenly distribute within a 1 cm^3^ tumor [7,58,59], which significantly minimizes the clinical utility of such therapeutics. Thus, creating convection force surrounding the tumor can improve diffusion and convection-mediated small molecule drug delivery. Additionally, convection created by the expansion and collapse of the SonoTran Particles bubbles likely increased the distribution of the large molecule Cetuximab within the tumor. This process was likely augmented by the reduced collagen matrix observed in the tumors following treatment, which is expected to further improve drug deposition. The reduction in collagen demonstrates the reduction in the extracellular matrix. However, it should be noted that other physiological properties, such as poor vascularization and high interstitial fluid pressure, can also impact drug delivery and were not directly evaluated.

Significant effort has been focused on improving drug delivery and distribution to benefit pancreatic cancer patients [60,61,62,63,64,65,66]. We described successful use of focused ultrasound, coupled with co-administered cavitation nuclei consisting of gas stabilizing SonoTran Particles, to improve the in situ deposition of clinically relevant drugs for pancreatic cancer. We can conclude that the innovative use of human pancreatic cancer xenograft in large animals will provide a highly effective and clinically relevant model for device development studies. Future studies will explore potential mechanisms to mitigate the off-target impacts of the treatment by optimizing treatment parameters; optimize the magnitude of drug delivery to targeted tumors; and evaluate the ability of this approach to impact morbidity and mortality. In conclusion, this study is the first to show quantifiable increases in the levels of both small-molecule chemotherapeutics and large-molecule antibodies delivered to pancreatic tumors in a large animal model of clinical relevance to human patients.

## Figures and Tables

**Table 1:** Extrapolating from clinical dose to experimental (piglet) dose.

Cetuximab Human Dose (250 mg/m^2^) [29]				
	Reference [30] Surface Area (m^2^)	Calculated Dose (mg)	Reference [30] Weight (kg)	Dose mg/kg
Human	1.62	405	60	6.8
Minipig	1.14	285	40	7.1
Micropig	0.74	185	20	9.3
Immune-compromised piglet Cetuximab dose used in this study				10.0
**Gemcitabine human dose (1000 mg/m^2^) [29]**				
Human	1.62	1620	60	27.0
Minipig	1.14	1140	40	28.5
Micropig	0.74	740	20	37.0
Immune-compromised piglet Gemcitabine dose used in this study				40.0
**Abraxane human dose (125 mg/m^2^) [31]**				
Human	1.62	202.5	60	3.4
Minipig	1.14	142.5	40	3.6
Micropig	0.74	92.5	20	4.6
Immune-compromised piglet Abraxane dose used in this study				5.0

**Table 2:** General characteristics of the animal, tumor, and treatment plan.

Pig ID	Number of Tumors	Tumor Volume (cm^3^)	Treated with SonoTran (Yes/No)	Drugs Injected
1	3	1.25, 0.7, and 2.5	No	None
2	0	NA	No	None
3	3	1.05, 0.7 and 0.54	No	None
4	2	1.0 and 2.1	No	SonoTran Particles
5	3	1.6, 1.09 and 1.25	No	SonoTran Particles
6	2	1.1 and 0.53	Yes	Gemcitabine, Paclitaxel, Cetuximab and SonoTran Particles
7	2	0.3 and 0.35	Yes	Gemcitabine, Paclitaxel, Cetuximab and SonoTran Particles
8	2	0.45 and 0.39	Yes	Gemcitabine, Paclitaxel, Cetuximab and SonoTran Particles

## Data Availability

The data that support the findings of this study are reported in the manuscript and in supplemental data. Any other relevant replicate or data are available on request from the corresponding author.

## Supplementary Materials

The following supporting information can be downloaded at: https://www.mdpi.com/article/10.3390/pharmaceutics15061585/s1, Supplemental Figure S1. Genotyping of piglets carrying modified *RAG2* and *IL2RG* genes; Supplemental Figure S2. MRM transitions and specific mass spectrometry tuning parameters for the quantification of gemcitabine and paclitaxel; Supplemental Figure S3. SonoTran-mediated cavitation treatment of pancreas tumors.
