# Supplementary material for: Improved Therapeutic Delivery Targeting Clinically Relevant Orthotopic Human Pancreatic Tumors Engrafted in Immunocompromised Pigs Using Ultrasound-Induced Cavitation: A Pilot Study
Source: Pharmaceutics. 2023 May 24;15(6):1585. doi: 10.3390/pharmaceutics15061585 (PMC10302458; doi:10.3390/pharmaceutics15061585)

Improved therapeutic delivery targeting clinically relevant orthotopic human pancreatic tumors in pig models using ultrasound-induced cavitation

Khan Mohammad Imran[1,2], Benjamin Tintera[2], Holly A. Morrison[2], Juselyn D. Tupik[2], Margaret A. Nagai-Singer[2], Hannah Ivester[1], McAlister Council-Troche[2], Michael Edwards[3], Sheryl Coutermarsh-Ott[2], Christopher Byron[4], Sherrie Clark-Deener[4], Kyungjun Uh[5], Kiho Lee[5] Paul Boulos[6], Cliff Rowe[6], Christian Coviello[6], Irving C. Allen[1,2*]

Supplemental data

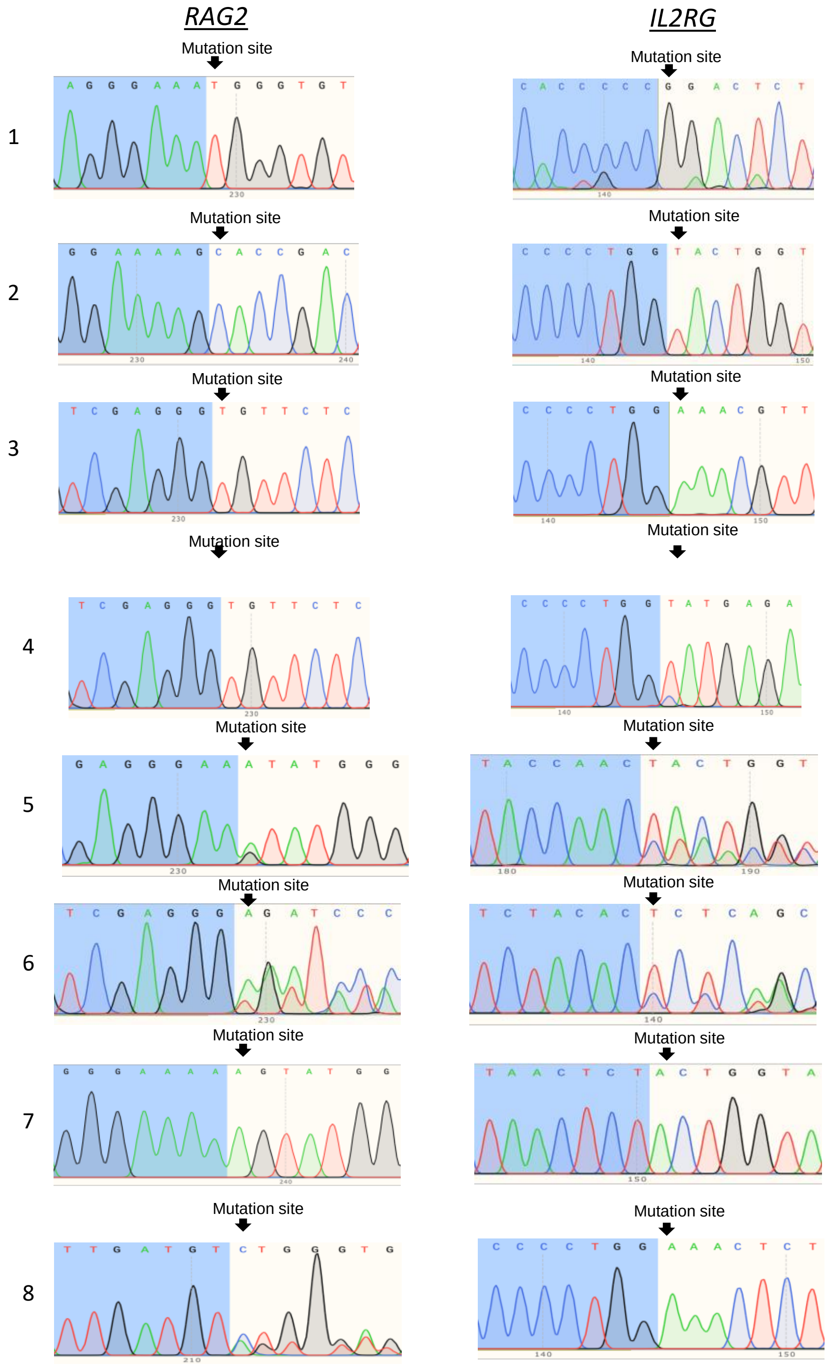

Supplemental Figure S1. Genotyping of piglets carrying modified *RAG2* and *IL2RG* genes.

Analyte	Parent Ion (amu)	Product Ion (amu)	Cone Energy (V)	Collision Energy (eV)	Quant/Qual Transition
Gemcitabine	264.0 $[M+H]^+$	111.9	38	16	Quantifier
	264.0 $[M+H]^+$	87.0	38	28	Qualifier 1
$^{13}C,^{15}N_2$-Gemcitabine (IS)	267.1 $[M+H]^+$	115.0	42	20	Quantifier
Paclitaxel	854.4 $[M+H]^+$	286.1	26	20	Quantifier
	854.4 $[M+H]^+$	569.2	26	13	Qualifier 1
Paclitaxel-d5 (IS)	859.9 $[M+H]^+$	291.2	26	20	Quantifier
	859.9 $[M+H]^+$	509.2	26	16	Qualifier 1

Supplemental figure S2. MRM transitions and specific mass spectrometry tuning parameters for the quantification of gemcitabine and paclitaxel.

Supplemental Figure S3. SonoTran-mediated cavitation treatment of pancreatic tumors.

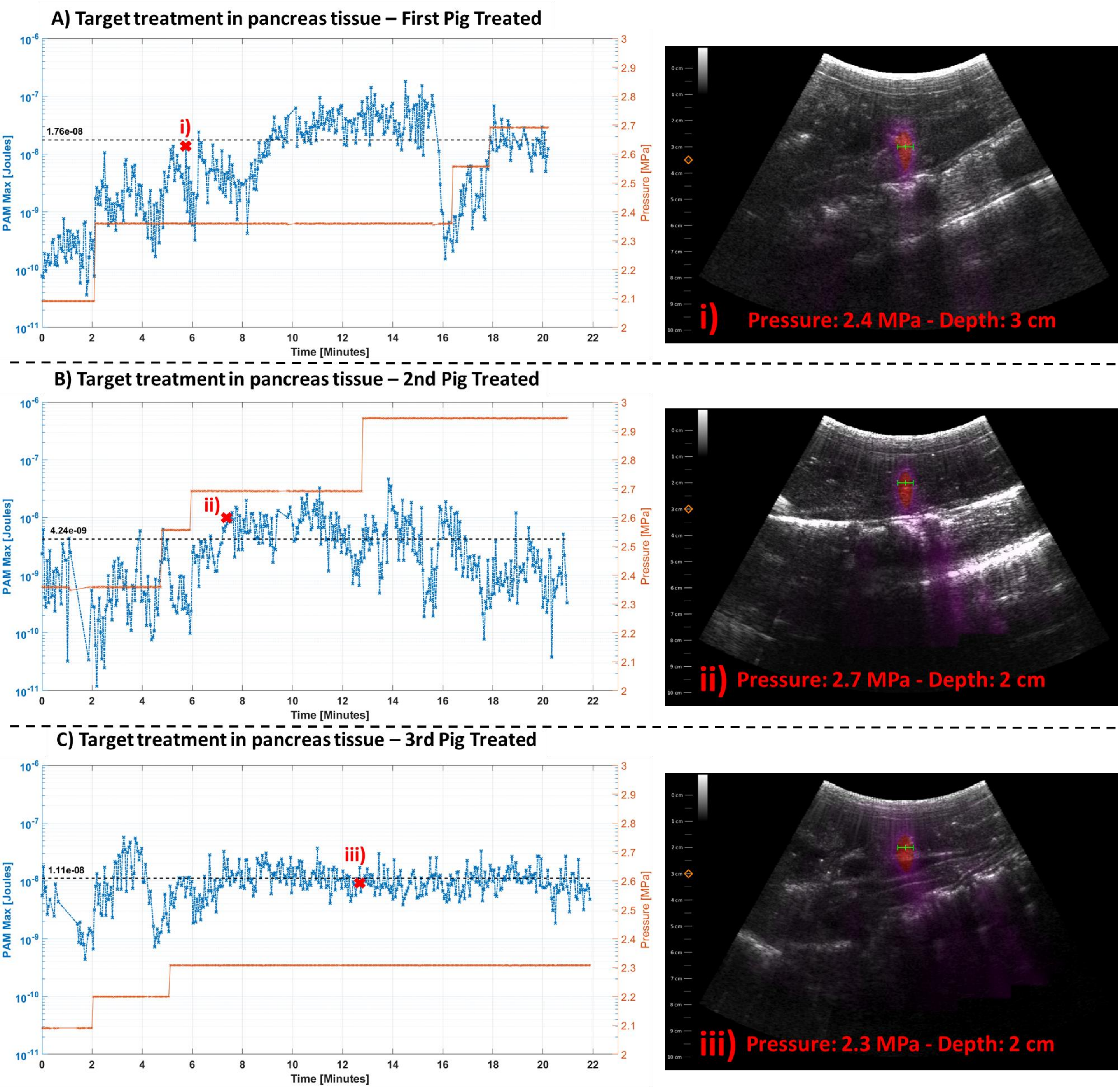

Supplement: Supplementary file 1 [file pharmaceutics-15-01585-s001.zip › pharmaceutics-2304261-supplementary.pdf]
